# Health workers’ experiences of implementation of Helping Mothers Survive Bleeding after Birth training in Tanzania: a process evaluation using the i-PARIHS framework

**DOI:** 10.1186/s12913-022-08605-y

**Published:** 2022-10-07

**Authors:** Fadhlun M Alwy Al-beity, Ulrika Baker, Deodatus Kakoko, Claudia Hanson, Andrea B Pembe

**Affiliations:** 1 Department of Obstetrics and Gynaecology, Muhimbili University of Health and Allied Sciences, 9 United Nation Road, Dar es salaam, Tanzania; 2 Department of Global Public Health, Karolinska Institutet, Stockholm, Sweden; 3 Department of Behavioural Sciences, Muhimbili University of Health and Allied Sciences, Dar es salaam, Tanzania; 4 Department of Disease Control, London School of Hygiene and Tropical Medicine, London, UK

**Keywords:** Helping Mothers Survive Bleeding after Birth, Competency-based training, In-facility training, Simulation, Peer practice facilitators, i-PARIHS framework, Postpartum haemorrhage

## Abstract

**Background:**

In-service training, including the competency-based *Helping Mothers Survive Bleeding After Birth* (HMS BAB) is widely implemented to improve the quality of maternal health services. To better understand how this specific training responds to the needs of providers and fits into the existing health systems, we explored health workers’ experiences of the HMS BAB training.

**Methods:**

Our qualitative process evaluation was done as part of an effectiveness trial and included eight focus group discussions with 51 healthcare workers in the four districts which were part of the HMS BAB trial. We employed deductive content analysis informed by the Integrated Promoting Action on Research Implementation in Health Services (i-PARIHS) construct of context, recipients, innovation and facilitation.

**Results:**

Overall, health workers reported positive experiences with the training content and how it was delivered. They are perceived to have improved competencies leading to improved health outcomes. Interviews proposed that peer practice coordinators require more support to sustain the weekly practices. Competing tasks within the facility in the context of limited time and human resources hindered the sustainability of weekly practices. Most health facilities had outlined the procedure for routine learning environments; however, these were not well operational.

**Conclusion:**

The HMS BAB training has great potential to improve health workers’ competencies around the time of childbirth and maternal outcomes. Challenges to successful implementation include balancing the intervention within the routine facility setting, staff motivation and workplace cultures.

## Background

Postpartum haemorrhage (PPH) occurs in up to 10% of women during childbirth and contributes to a quarter of maternal deaths [1, 2]. Clear recommendations for preventive intervention to reduce PPH are established [3, 4]; however, adherence to these guidelines is limited, especially in low and middle-income countries [5, 6]. Consequently, high PPH morbidities and mortalities persist in many countries, Tanzania included [7–9].

Different maternal health interventions include in-service-trainings to improve health workers' adherence to evidence-based guidelines; however, evidence of their effectiveness is limited. A recent review proposed that training combined with other strategies has some potential to improve health workers' practices. Thus, multifaceted intervention may lead to higher effects. Evidence is, however, limited on why some interventions have resulted in impacts while others do not [10, 11].

Implementation science systematically addresses why and how some interventions lead to better uptake of knowledge into routine clinical practice while others do not [12]. For example, when implementing new interventions in maternity health services, one must carefully consider the target's understanding, acceptance, motivation, interactions, and available support to facilitate uptake. Moreover, a clear understanding of the context or work environment and how rigid or flexible to allow necessary changes to support the success of an intervention [13]. Several implementation theories are used to understand, describe and explain an intervention, what went according to plan and whether and what context-specific adjustments were made [14–17]. Such theories can explore and document feasibility, acceptability, fidelity, barriers, and facilitators of an intervention uptake. Using implementation theories in the design and planning of healthcare interventions may also improve the uptake and effectiveness of such interventions [18].

One such implementation theory is the "Integrated Promoting Action on Research Implementation in Health Services (i-PARIHS)". This determinant theory explores the interaction of an innovation or new intervention, the *work environment, the* targeted group and the facilitation of the intervention, describing the path to an intervention's success or failure. This framework has been used before in planning maternal health interventions [19–21]and also used to organize the analysis of process evaluations of some interventions [17, 22].

In 2015, we conducted a trial of the effectiveness of the Helping Mothers Survive Bleeding after Birth (HMS-BAB) in-service competency-based training on provider skills, improved clinical practices and improved health outcomes [23, 24]. The HMS BAB in-service competency-based training uses a mix of theory and practical simulation sessions during a one-day training session with all cadres working in the maternity ward: medical doctors, nurse-midwives, non-physician clinicians and auxiliary nurses. The implementation was done step-wise, where external facilitators trained all maternity ward providers during one-day sessions. In addition, external facilitators identified and coached a pair of qualified providers as peer-practice coordinators. The role of peer-practice coordinators was to lead their peers on skill drills within the labour ward weekly. The drills/practice sessions are to augment skills acquired during initial training. External facilitators supported peer-practice coordinators through phone calls but not on-site. No additional resources in terms of drugs and medical supply, infrastructures, or human resources are part of the intervention.

The positive findings of the HMS BAB intervention's effectiveness stemming from our cluster-randomized trial [23] prompted a further need to understand why it worked. In the current study, we explored health workers' experiences of the HMS BAB intervention implementation process: what worked, what did not, and how it achieved the intended outcomes. Findings from this study can support the development of future training in general and also provide hints on what to emphasize for further scale-up.

## Materials and methods

### Study design

This study was a qualitative process evaluation study of the HMS BAB training intervention using the i-PARIHS framework. We used this framework during analysis as it resonated with our main findings.

The i-PARIHS framework has four main constructs: *the innovation*, *the recipients*, *the construct* and *the facilitation *[12, 14, 25]*.* The innovation construct looks at the inherent characteristics of innovative intervention that make it more or less appealing and how it compares to similar or available interventions. The facilitation constructs examine the features and abilities of the recipients and the context where the innovation was done [21, 25]. Facilitation refers to the actual delivery of the intervention either by an external or an internal facilitator [25]. It is an essential construct and may influence the acceptance and uptake of the new knowledge into routine care. The last construct: the “context”, refers to the environment, including the local working condition. It maps how the intervention interacts with the facility organization and the more extensive health system. The “recipients” are the target group, thus the health workers; their professional background and identity determine the process and outcome, their interactions with the innovation and the context. Table 1 shows the different constructs and their characteristics.

**Table 1:** HMS BAB Training Intervention description using the four constructs of the i-PARIHS

Construct	Description of the construct in the HMS BAB training intervention
Innovation	Competency-based in-facility training, use low-fidelity birthing simulator: Mama Natalie. Inclusive to all maternity-ward staff (multi-profession) Training curriculum covers communication with pregnant woman and family, standard delivery, Active Management of Third Stage of Labour (AMTSL), assessment of excessive bleeding, care of newborn, and preparation of advanced/referral care when needed Graphic flip chart, learner’s guide, posters
Facilitation	Short theory sessions followed by role plays, case scenarios and skill practice on the Mama Natalie simulator. Practice sessions are followed by debriefing and group discussions. Some training materials were available in local language, Kiswahili Outside/district facilitators- pairs of outside facilitators conducted initial in-facility training in all the facilities, they identified and coached local facilitators or “peer practice coordinators.” The “peer practice coordinators” organised colleagues for short weekly practice drills on PPH-specific scenarios
The Recipients	All maternity ward health workers of different cadres trained on the initial day, used the knowledge and skills gained and continued to practice with simulators
Context	Training is in-facility, using the local environment and managing everyday daily tasks and creating safe learning and regular practice sessions at the workplace

### Facility and participants selection

The main HMS BAB trial, where the current study was nested in, was implemented in two regions of Tanzania; the northern part or lake region and the southern regions. For the present study, we selected four districts, two from each region. Each district had two-level health facilities: a district hospital and large health centers. Within the health facility, we selected health workers to achieve variability of participants who (1) received the HMS BAB training and participated in the weekly drills, (2) had the role of peer practice coordinators and (3) started to work in the maternity wards after the intervention period.

As the study was nested within the HMS BAB trial, the good collaboration had already been established with the district health administration facilitated health workers’ willingness to participate.

### Data collection

Data collection was done between March and April 2017, a year after implementing the HMS BAB intervention. The leading researcher and two social scientists used a pre-prepared topic guide to facilitate the FGDs.

We conducted eight Focus Group Discussion (FGD): one per health facility level per district, each had 5–7 participants. Getting all cadres in all FGDs was challenging; for example, one FGD had only auxiliary nurses, a medical officer and a non-physician clinician. Reasons for non-availability of staff included attending out-of-site training or not having other cadres.

We used a pre-prepared topic guide to explore health workers’ experiences. The tool had questions on the unique characteristics of the training and their experiences of what worked or did not work well. Furthermore, health workers were asked to highlight needed improvements if the intervention was to be scaled.

The health facility manager assisted in identifying and informing participants a day before the FGD. The FGDs were scheduled and done during the work shift-exchange, to increase the number of participants from both morning and afternoon shift. The discussions were done within in a quiet room/space within the health-facility to ensure privacy and minimize interruptions. All FGDs were done in the local language Kiswahili, and were audio recorded. Participants used codes rather than names or cadre. Additional checklist was used to collect information like age, experience, education and cadre. On average, each FGD session lasted for 60 min. The recorded discussions were later transcribed verbatim and translated into English. The lead author performed a quality check in half of the translated transcripts. All transcripts and field notes were de-identified and secured safely.

#### Ethical considerations

The study was reviewed and granted ethical approval from the Muhimbili University of Health and Allied Sciences Institution Review Board. All study activities observed national research protocols. All participants were informed of the study, agreed to participate, and gave written consent.

#### Research team

The lead researcher, FAA, an obstetrician from a tertiary teaching hospital, organized the research activities and facilitated three of the eight FGDs. FAA was part of the HMS BAB trial team but had few interactions with HMS BAB trainers. Two research assistants participated in data collection; they were social scientists and experienced in qualitative data collection. They served as field note taker, recorder, and assistant facilitators. The two qualitative researchers had no prior contact with the study participants.

#### Analysis

We used deductive content analysis [28] informed by the i-PARIHS framework of intervention implementation. All translated transcripts were entered in an analysis software program *MAXDAQ 2018* for analysis. All transcripts were read and re-read by three* authors to familiarize them with the text. The first author developed a structured analysis matrix with inputs from the second author. Paragraphs were fitted into the structured matrix that consisted of the main i-PARIHS constructs, and its categories the training is the “innovation”, “facilitation” is how the training was delivered to hasten uptake, both the first-day training and the following practice drills on weekly practices, “recipients” are the health workers including local peer practice facilitators, who received the training and continued to practice the drills and “context” is the overall organization structure that facilitates or hinders implementation and uptake of the intervention. (Characteristics of the constructs) [27]. Data was then condensed into meaning units, coded, and later grouped into sub-categories. Some of the sub-categories were reflected in more than one construct and not all characteristics of the constructs were present in all.

## Results

A total of 51 health workers were included with an average 5–7 per FGD. These were medical doctors (2), non-physician clinicians (2), nurse-midwives (35), auxiliary nurses (11) and nursing assistants (1). The median age was 34 years (range 23–50 years), the majority were females (80%, 45/51).

**Fig. 1:**
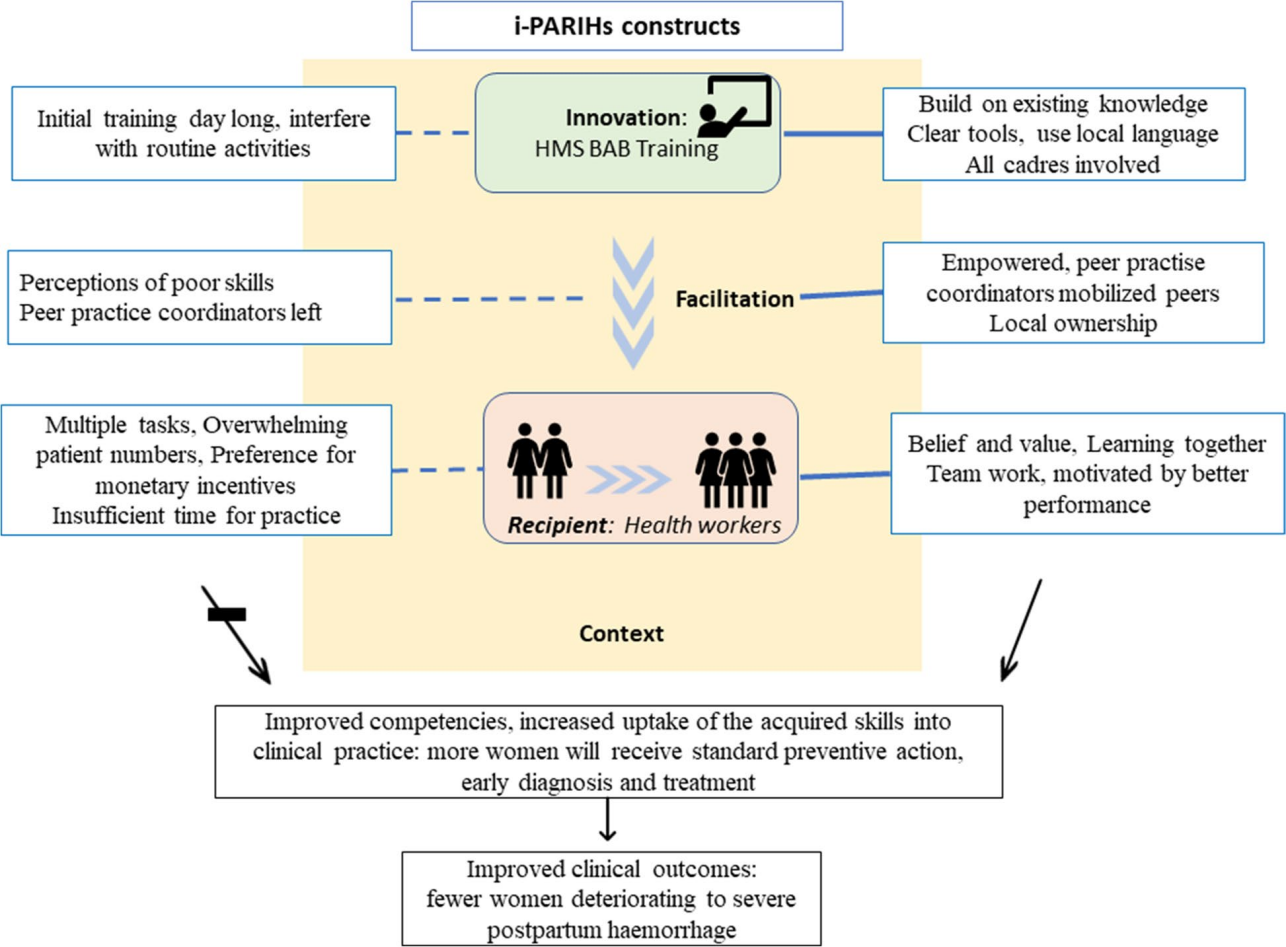
Schematic model of the barriers and facilitators presented using the i-PARIHS framework

We present the results in four constructs of the i-PARIHS: innovation, recipients, facilitation and context, as shown in Table 2. We present individual constructs as best as we could; however, there was strong interdependency between the constructs. Figure 1 shows schematic representation of the constructs.

**Table 2:** Themes, categories, and sub-categories

Theme	Category	Sub-category
**Innovation**	Degree of fit	Training was basic
		Training refreshed what we already learnt
	Clarity	Local language facilitated learning
		Well-designed clear guides and SOPs
		I carry the book everywhere
	Usability	We detect and manage PPH early
		We know what to do
	Observable results	We remind each other of steps
		Now we prepare for PPH
		We have less PPH complications and deaths
	Relative advantage	All cadres benefitted from training
		Training was more practical than theory
		One day is too short
**Recipients**	Beliefs and values	Training makes us do better
		People have different obligations and commitments
	Motivation	Incentives are important to all
		We get vitamin A when we go to out-of-facility training
		Money is not always necessary
	Time, people and tasks	We use our own time to practice
		It is hard to do weekly practices
		We are too few to practice
	Teamwork and collaboration	We train new members and teams
		We give each other training feedback
		Peer practice facilitators left without handling over
**Facilitation**	Ownership and participation	It is hard to get everyone to practice together
		Regular practices enhanced learning
	Empower	Mentoring is a big responsibility
		Now I trust myself more
		Peers enhanced learning
		Now we do things properly
**Context**	Learning environment	Clinical obligation affect concentration during training
		In-facility training should be better planned
		Good to learn in your environment and with your peers
	Workplace culture	New peer practice facilitators needed to be trained after rotation
		Training was timely after rotation
		We routinely discuss our practices and deficiencies
	Learning networks	Out-of-facility networks are lost

### The innovation: the HMS BAB training with the eight-weekly package

#### Degree of fit: training contents fit with existing standards

Health workers described the training components, the training's aims and coverage, and why they thought it was important. They described training that covered basic delivery skills, early detection and management of PPH. A reminder of what they had learned during their nursing education. Furthermore, additional components for newborn care, respectful maternity care and organizing for a referral to advanced care were well received.

*“…We learned these things in nursing school. It is not that they were new; the training came to remind us practically.”* P4: enrolled nurse-midwife, health centre, FGD4

#### Clarity: Training well designed-tools and delivered in an easy-to-follow manner

Health workers described well-designed easy-to-follow training tools in their familiar language that enhanced training. The training materials and use of simulators for scenario-based practice improved the understanding. Lower cadres, who did not have prior clinical training, could follow the training and understand the required standards.

*“In our facility, we have the HMS posters on the wall of both languages English and Kiswahili: the posters direct you on everything important that is supposed to be done when you encounter PPH.”* nurse-midwife, health centre, southern zone, FGD4

#### Usability: we acquired the knowledge and skills needed for PPH

Health workers perceived to have acquired improved competencies in diagnosing and managing PPH following the simulation and practice-based training. They perceived that they were more vigilant in identifying excessive bleeding and could give initial management, such as adding intravenous oxytocin. Some nurse-midwives admitted that they did not know or practice essential interventions such as repeating intravenous oxytocin for uterine atony.

*“Before the [HMS BAB] training we had difficulties. Now I can detect specific cause of bleeding: whether there is a tear, a retained placenta or uterus failed to contract…after the training we know what to do. You go stage by stage”* clinical officer, health centre, lake zone FGD6

#### Observable results: we observe less cases of severe PPH

Most health workers reported that they feel much confident of their ability to recognise and manage PPH. They start management early and that many instances they have arrested bleeding before additional help arrive and hence fewer severe PPH cases are reported.

*“There is a difference because I see PPH that occur now days are not like the ones in the past. We had a lot of PPHs and maternal deaths due to PPH. Currently, I can say that we comparatively have fewer cases…”* enrolled nurse-midwife, health centre, hospital, southern zone, FGD1

#### Relative advantage: the training was short and inclusive of all cadres

The training was non-discriminatory and useful to lower cadres who usually are left behind during in-service training. In-facility training also means a larger proportion of staff are aware of the training, are trained or will know about the training and join some of the sessions. Usually, in-service trainings are done out-of-facility, only one or two people attend due to costs and the rest of staff get training or feedback from those who attended out-facility training. Most of the times those left behind do not benefit from feedback sessions.

*“I liked this training that came to our place of work… they met us: health workers who are giving care to clients. When they call people away from their facilities there is always a bias regarding who attends.”* enrolled nurse-midwife, district hospital, lake zone FGD6

Health workers perceived this training to be advantageous as it was more practical, with all staff getting time to practice on the simulators. However, the initial day was long as all had to practice. Consequently, some health workers felt overwhelmed on the first day with possibilities of being confused or mixing up what they had learned.

*“The training was practical and felt real and hands on, because Mama Natalie (the simulator) is real, we practiced on the simulator. This is different from sitting in a classroom listening to theory session. We practiced several times. We practiced third stage of labour, and the facilitator was there to give you feedback at the end and make you realize your skill gaps.”* nurse-midwife, health center, southern zone FGD8

### Recipients: health workers beliefs and capacity in carrying and receiving the intervention

#### Belief and values: health workers believe their work is important

Health workers believed working in the maternity ward is a call. They recognized a need to have regular updates and are committed to learn and improve their competencies. Furthermore, they were mindful of capabilities and commitments levels amongst themselves. Health workers realized they had an obligation to the community and to the health facilities to provide the best possible care.

*“We need trainings for different things, because we believe we are giving important service here in the labour ward, nowadays the biggest campaigns are on reducing maternal deaths, and neonatal deaths”* nurse-midwife, district hospital, lake zone, FGD6

#### Motivation: health workers motivation affect performance and sustainability of an intervention

Health workers discussed their experiences and expectations from this training. While they were happy with the in-facility training, they expressed that they expected some monetary incentives for participating especially peer practice facilitators who took time to organise and lead the weekly practices. That lack of such incentives may cause non-adherence to regular implementation of the intervention.

*“There should be motivation and recognition to those who continue with the weekly practice sessions. Because, after their routine duties, they organise and lead people to learn and practice…so we need to recognise them, so that they continue to work diligently.”* nurse-midwife, female, health centre, southern zone, FGD2

Some however thought that acquiring skills and competencies was enough motivation and were ready to do the regular practices and coach their colleagues without monetary incentives.

*“That is how we come back and say those who are committed and motivated will do. If you are moved by something you will not wait for payment. Because every time you teach you gain more”* nurse-midwife, health centre, southern zone FGD4

Some participants appreciated that local mentoring and coaching of local facilitators was good, however, it is better to get such a person more training even send her out of the facility for more skills but also for more accountability as the below quote demonstrate.

*“But one person should also go out for supervisions and teaching training. Taking someone here to do mentoring is not taken seriously. But if you have gone out of here and have enjoyed some money then you are obliged and committed to do it.”* clinical officer, district hospital, lake zone FGD 5

#### Time, people, and tasks: health workers sometimes unable to balance routine duties and practice sessions

Health workers perceived that they should be informed on the training well in advance, some knew about the training on the same day, and some had continued for the training day. Also, the initial one day was not enough, especially because many trainings started late during the day, and in hospitals with more health workers getting all to practice within working time was difficult. Health workers advised to have more training days or to health workers train in groups.

Furthermore, health workers perceived the half-day used for coaching peer practice facilitators was insufficient, and that they would benefit for more extended training on technicalities of the simulator and training materials. The intervention required that peer practice coordinators organize and lead their peers in short practice drills on weekly basis. Such required time and planning and health workers expressed difficulties in arranging this. Consequently, few health workers attended practice, or few practice sessions were organised. After the mandatory eight weeks, the practice sessions were reduced.

*“There is an issue of time, the weekly practice means time consumed. here are activities and duties for almost every time. We have many duties. So, it is not easy to sit together and practice with Mama Natalie. You can’t even say that you will practice in the morning, there are more duties then, it is impossible”* nurse-midwife, district hospital, lake zone, FGD5

#### Collaboration and teamwork: we train and work as teams

Despite the time constraint, some recipients perceived the training techniques and practiced regular drills has augmented the working and training together culture. Peer practice coordinators and those who received training make additional efforts to train those not trained or newly assigned maternity ward staff.

*“Because there are many people who were trained here in the labour ward, and Mama Natalie is here. We prepare a day with the in-charge so we can practice and remind each other and train the new team who did not attend the initial training”* nurse-midwife, district hospital, southern zone, FGD1

### Facilitation: the initial training and the weekly-practice sessions by peer practice facilitators

Health workers liked the district HMS BAB trainer’s facilitation and expressed that it was an efficient and hands-on training that enhanced their learning and practice. They all had several practices and felt they had t like they mastered the skills. The health workers also liked that a few were selected and trained as peer practice facilitators to lead the local practice and that they all had access to the materials and simulators. The peer practice facilitators were helpful in the learning process.

#### Enabling and empowering peer practice facilitators

Health workers perceived that being a peer practice facilitator was a big responsibility and although they received initial support, it took them time to master the mentoring skills. In their views, these local facilitators should even get outside facility training to master more mentoring and coaching skills. As a result of these regular practice sessions, they have all acquired skills and are quick to correct each other whenever they make a practice mistake. This was demonstrated by the following quote.

*“After the initial training, we were trained as peer practice facilitators. I am also a facilitator for another training, where we took five days to teach our colleagues, the nurses. … This (HMS BAB) flipchart is lengthy; I see some challenges using it during the training…”* nurse-midwife, peer practice coordinator, health-centre, southern zone, FGD2

Most participants described there was always a challenge to get practice time balanced with their routine duties given the human resource shortages within the facilities. Many expressed that it was difficult to get everyone into the practise sessions and that organising several practice sessions is a challenge.

#### Ownership and participation by those responsible for change

Health workers described that the weekly practices were very valuable to maintain their skills. Majority explained that they diligently met weekly and practised for the period of the intervention. After this period, it became harder to meet weekly and would meet once or twice in a month.

*“They should do it every week, and they did initially, but now I see like they are tired. In the beginning every week we practiced before leaving the shift.”* Nurse-midwife, district hospital, southern zone FGD3

Health workers also described they now have integrated the training and make sure that each new team that comes to work in the maternity ward is trained.

*“We do that (mentoring) …we have forgotten the last time we got severe PPH. it (mentoring) has become continuous and I feel that almost all of us were mentors.”* nurse-midwife, peer practice coordinator, health-centre, southern zone, FGD2

### Context

#### Learning environment

Doing the initial training in-facility was a positive experience. More health workers were trained, hence reduced need for feedback sessions from few members who attended outside training. Generally, health workers perceived that it was good to learn in their own space with regular colleagues. The on-site training reduced potential absenteeism from staff attending out of facility training.

Nevertheless, the facility setting was not always ideal to training as other activities are on-going. As the training was on-going, some had to stop and attend patients, and few missed the training as they had to stay and give service. Some health workers even preferred out of the facility training in order to minimize disturbances and not be interrupted when they are being trained as it happened during this training.

*“Because (out of facility training) there is no interference, you cannot participate well, at the end of the day we say that central training may be better than on-site training. Because there is no interference, we are strict and nobody knows you there (in the central training) to give another task beside the training. There are no movements in and out of training.”* nurse-midwife, district hospital, southern zone FGD8.

Some health workers perceived that they were not well informed before the initial training. Some felt obliged to continue with the training even when they came from a night shift as they did not want to disagree with their supervisors. Health workers advised future trainings to be done smaller groups for better organization and outcomes.

#### Workplace culture and practices

Most facilities had a system in place that promote learning and sharing of information during clinical meetings and morning reports. Health workers discussed the potential of such meetings to be used as practice sessions.

*“In the morning report we discuss what happened the day before, admissions, women in labour, normal deliveries and challenges encountered during your shift such as stock-outs and shortages. Everyone says report his/her challenges”* nurse-midwife, health centre, southern zone, FGD4

Health workers had mixed perceptions of existing workplace culture of rotation: allocating health workers in different departments. This had some implication for the training especially on planning and conducting the weekly practice session. In one facility, both peer practice facilitators were moved immediately after the introduction of the intervention and new peer practice facilitators had to be re-trained.

*“What happened was one of the two peer practice facilitators had a transfer to another facility. The second person went to school. When both left, they had not trained anyone or handed over the Mama Natalie. It was only locked in the closet…until they (district trainers) came and trained new peer practice facilitators.”* nurse-midwife, district hospital, lake zone, FGD5

#### Learning network

As more in-facilities trainings are done, the learning networks with other colleagues from other facilities may be lost. Health workers felt it is important to keep in between facilities relationships and learning open which also aids communication when they refer patients in between facilities.

## Discussion

We systematically document health workers' perceptions and experiences towards the HMS BAB training using the i-PARIHS framework: innovation, recipients, facilitations, and context. Health workers highlighted their satisfaction with the design, content and tools, the delivery technique, and the practice-based sessions and debriefing opportunities. They also appreciated the on-site training that included all. Weekly practices stimulated a learning culture within the working environment. The recipients acknowledged the need to update their clinical skills regularly. Providing additional support to peer practice facilitators and mentoring to manage competing tasks in the facilities would improve the training.

### The innovation: a potential intervention for sustained change

The training covers essential skills required during childbirth delivered on-site to all staff compared to the traditional out-of-facility training: i) no travel, ii) no perdiem costs, iii) less staff absenteeism and iv) involvement of all cadres. The peer practice concept empowered staff long-term, and built capacity without jeopardizing patient safety through absenteeism. Thus, the HMS BAB intervention has great potential to address the existing workforce shortages and improve competencies. Notably, health workers acknowledged and accepted the training methodology and usability.

### Innovation and facilitation: facing realities

Health workers' experiences indicated that the delivery of one-day training to all providers clashed with the realities disrupting clinical care and routine activities. They further advised integrating weekly practice sessions within the existing facility routines, balancing challenges of staff shortages, multiple sessions and competing tasks. There was also a mismatch of cadres who should provide care and who were providing care within the maternity ward.

Furthermore, integrating training interventions with other quality improvement approaches such as near-miss audits and critical reflections will accelerate the uptake in real-time.

### Facilitation: barriers and potential for improvements

The use of realistic simulated scenarios, debriefing and reflective learning sessions and teamwork during the HMS BAB intervention had a positive effect [26, 27]. Local facilitators empowered local staff and improved their ownership and commitment. Selection of local peer practice facilitators requires local inputs and should not base on performance during training or popularity. Instead, a dialogue with the facility to identify individuals' characteristics that make them strong candidates for the role of a peer practice coordinator, namely communication and leadership skills and commitment. Local facilitators demonstrated resilience and flexibility in planning and conducting the weekly session, negotiating a balance with their colleagues. Individual facilitators' characteristics, such as flexibility, assertiveness and conflict resolution skills improve the uptake process [18, 28]. In the future, we think careful selection and investment in these leaders and empowering them with clinical and leadership skills can increase the long-term effect of the training [29–31]. We also that there may be difficulties to identify these individuals due to different workplace dynamics and a local dialogue in the process is essential.

### Recipients: the central role of the health workers

The intervention requires health workers to commit their time and participate actively in the initial training and weekly practice sessions. Evidence suggests that the dire understaffing in facilities and high delivery volumes overwhelm providers [32, 33]. In such conditions, we applaud health workers for their resilience and hard work and for being the drivers of change. For the scale-up of the HMS BAB or similar training interventions, careful planning should balance the extra efforts to avoid overwhelming trainees and peer practice coordinators.

Integration of the training with the continuous professional development points may help providers invest their time in practice sessions [34–36]. Awarding these points may also reduce the need for or insistence on monetary payments given during training, reducing the cost.

### The context: a facilitator and barrier to implementation

Several contextual factors posed potential barriers to the training and future scale-up. Most facilities had a rotation culture, moving those already trained, including peer practice facilitators, to other departments – a common practice [13, 37]. Also, health worker shortages and shortages of medical supplies have been described [11, 33].These implementation challenges may also partly explain the limited – albeit positive – effect of the intervention observed in the main trial [23].

This study further highlights the HMS BAB training potential in improving health workers’ proficiencies around childbirth and maternal health outcomes also supported by others [38]. We also highlight several challenges that need to be tackled to improve implementation and overall uptake of this type of training.

### Methodological considerations

Our tool was designed to capture barriers and facilitators of the intervention process but was not structured according to the i-PARIHS framework. However, recent literature on this framework reported that the i-PARIS framework is commonly used to organize the analysis process [17, 19, 21, 22]– thus our limitation is common. We appreciated during the analysis that the i-PARIHS constructs were easily applicable to our data, underscoring the relevance of the framework to highlight critical facilitators and barriers of interventions. Still, we may have excluded information that did not fit the predetermined categories during the analysis. Apart from the first author, three other authors participated in the initial analysis with discussions. We believe that we included all essential components in the analysis. There were also several instances where the data fit in more than one construct. We tried as much as possible to group such information to the most fitting construct.

We only explored the perspectives of the health workers and no other groups like managers. Health workers were the main target of the intervention, and we believe they had the relevant knowledge of the implementation process. We had a mixed participant group, and we believe they shared enough experiences to fit the framework. The study was done a year after the main intervention, thus could have resulted in some recall bias.

Other components of the implementation process were not assessed, such as fidelity, acceptability, and cost analysis which would have improved the findings; due to time limitations as the main HMS BAB trial had been completed.

## Conclusion and recommendations

Overall, the HMS BAB training intervention was well d positively embraced by the health workers. They appreciated the content, the delivery, the learning and what they perceived as immediate results from the training. More careful planning of when and how to deliver the intervention is needed to improve the intervention and avoid interruption of the clinical processes. Furthermore, integrating the training into continuous professional development and assigning credits may help health worker motivation. We believe our analysis highlighted important insights and indicated the value of process evaluations alongside effectiveness evaluations.

## Data Availability

The dataset used and analysed during the current study are available from the corresponding author on reasonable request.

## Abbreviations

AMSTL: Active Management of Third Stage of Labour
DMOs: District Medical Officers
FGDs: Focus Group Discussions
HMS BAB: Helping Mothers Survive Bleeding After Birth
IRB: Institutional review Board
i-PARIHS: Integrated Promoting Action on Research: Implementation in Health Services
PPH: Postpartum Haemorrhage
MoHCDGEC: Ministry of Health, Community Development, Gender, Elderly and Children; WHO: World Health Organization
